# Case of Tertian Intermittent

**Published:** 1828-04

**Authors:** H. R. Oswald


### TERTIAN INTERMITTENT.

*Case of Tertian Intermittent.* By H. R. Oswald, Esq.

January 12th, 1828, 2 p.m.—Hugh Caton, ætatis twenty-one, mariner; is shivering in the cold stage of an ague, which proves uncommonly severe. Has been subject to fits of the same kind since July last, at which time he had a fever at New Orleans, from which he did not recover during the voyage home. The fits used to come on at irregular intervals, generally once a week; but since he arrived at Liverpool, two months ago, they have attacked him regularly every second day, between this hour and mid-day. The general duration of the cold stage is from two to three hours, after which it is succeeded by a hot dry fever for three or four hours more; and the fit passes off towards midnight by sleep and perspiration. During the intermissions, his appetite and other functions are tolerably healthy; but he never feels so well as to be able for his duty. His complexion is pale and yellow, but, though thin, he is not emaciated. Has used no remedy except chewing occasionally a little rhubarb.

As the history of this case is its principal novelty, and appears to me unusually interesting, I shall take the liberty of giving it a good deal in detail. From cross-examination, and other credible reasons, I have no doubt the following is correct.

On the 12th of April, 1827, Caton sailed from this port in the brig Hardwell, of Ayre, James Weddell, master, and ten men, with a general cargo for the Gulf of Mexico. They made the River Tampico, which they entered about the 3d of May, all well. When at anchor in the bay, lightening the cargo, three or four days after their arrival, the master, James Weddell, was seized, about two p.m., with great pain or cramp in the stomach. About three p.m. had incessant vomiting, and, after a severe night's suffering, died the next day at noon. The master was said to have died of the black vomit. He had been on shore upon business, but only for a few hours at a time. About the middle of July, the vessel sailed for New Orleans, in ballast of dry white gravel and sand; and arrived there all well after a week.

They lay three weeks at New Orleans, abreast of the market-place, and near banks of mud, gravel, and hard clay. During the first week, the mate and the second mate, and a lad sixteen years of age, were taken ill of what was called there "the fever." In about a week, all three were able to be on deck again. In the second week, my patient and three other men, Brown, Hansel, and Newton, were sent down the river in a boat to fetch the hawser, which, from the brig getting foul of a steam-vessel, in coming up, had been left on the banks of the river. They left the vessel about nine P.M., and were out all night at work, in consequence of the hawser having become intricately entangled amongst the trees and mud on the margin of the river. They had a hard row up to the town next morning, under a burning sun. All the boat's crew began to feel themselves fail very much after the sun got up, so that they were hardly able to row the boat back again to the vessel; and, when they did arrive, were unable to put the hawser on board. Headache came on about noon, with overpowering lassitude. Soon after my patient passed into a high fever, during which he cannot describe his symptoms, excepting the severe vomiting, with pain in his right side. After about a week, the fever began to abate; but he continued subject to shiverings occasionally.

Brown, Hansel, and Newton, and subsequently another man named Kaye, were sent ashore to the hospital, whence, except Hansel, they never returned. Knows not the reason he was not sent ashore with them, except that he was considered not so ill, and at the time none of the crew were well, although most of them were able to move about on deck. They all had headache and vomiting, and some of them were yellow. All those that recovered became yellow as the first violent onset of the disease began to subside. My patient is still very pale, and of a dingy yellow complexion.

After remaining at New Orleans three weeks they left it, when not one of the remaining part of the crew was in proper health. After getting into the Gulf of Mexico, they remained a month at Cayes West, in consequence of the vessel running aground. Two more of the men went ashore at this place, where he supposes they died. But, notwithstanding this delay, those remaining on board did not recover, but continued quite unfit for duty all the voyage home. The vessel was navigated home by the crew of a vessel lost in the Gulf, which they took on board at Cayes West. None of this fresh supply of men had any sickness in the passage. The old crew, five in number, continued subject to cold fits, pains in the sides, swelled legs, lassitude and debility. The carpenter, in particular, suffered from erysipelas in his right ankle, and went into the infirmary when they arrived at Liverpool. My patient distinctly states he had a severe rigor once a week, generally on Sunday; and that the disease did not assume the tertian type till his arrival at Liverpool and this island in the beginning of November last; but more especially after exposure to wet, when he first returned to Douglas; since which time the fit has come on regularly every second day about two o'clock.

After evacuating the contents of the stomach and bowels well, he was directed to take a teaspoonful of the Bark every three hours. The approach of the fit was also checked by a dose of Tinctura Opii; and after ten days the cure was complete.

*Remarks.*—I shall refrain from entering into a recapitulation of the authorities which suggest the probability of an affinity existing between some of the fevers of the old continent and those of the new. The opinion that affinities do exist between many diseases apparently dissimilar has gained ground lately, but it is by no means a new doctrine. The foregoing statement furnishes direct evidence, pointing us to reflect on an affinity between the fever of New Orleans and the intermittents of our latitudes. If we could be quite certain that this case of ague did not originate after my patient's arrival in England, we have a demonstration, almost irrefragable, that such an affinity actually does exist; for in it we distinctly trace the fever of New Orleans, (the yellow fever,) passing into the ague of Europe, merely from the change of climates and the operation of time.

But if the history of the crew of the Hardwell is not considered sufficient evidence that the disease under which my patient laboured in the Isle of Man originated in the River Mississippi, but came on from an exposure to a new cause after his arrival in England, the foregoing statement at least illustrates the history of both ague and the yellow fever, in so far as it proves that the latter increases the susceptibility of the human body to the attacks of the former: a circumstance opposed to the doctrine of dissimilar diseases being antidotes to the attacks of each other in general. It may also be questioned whether the fever which my patient had at New Orleans was the yellow fever, or simply a remittent? The reply must rest on evidence upon which controversialists in the philosophy of medicine would not, perhaps, implicitly place much confidence,—viz. on the account of a young man given from memory. But, though a novice in such matters, many of the principal symptoms of the first attack are stated sufficiently correctly to warrant us in considering the disease which killed so many of the crew of this vessel, and disabled the whole of them, to have been no other than the yellow fever.

*Douglas, Isle of Man.*

